# Impact of a VA–ECMO in Combination with an Extracorporeal Cytokine Hemadsorption System in Critically Ill Patients with Cardiogenic Shock–Design and Rationale of the ECMOsorb Trial

**DOI:** 10.3390/jcm12154893

**Published:** 2023-07-25

**Authors:** Franz Haertel, Thomas Lehmann, Tabitha Heller, Michael Fritzenwanger, Ruediger Pfeifer, Daniel Kretzschmar, Sylvia Otto, Jurgen Bogoviku, Julian Westphal, Christiane Bruening, Thomas Gecks, Mirko Kaluza, Sven Moebius-Winkler, P. Christian Schulze

**Affiliations:** 1 Department of Cardiology and Intensive Care, University Hospital Jena, Am Klinikum 1, 07747 Jena, Germany; 2 Center of Clinical Studies, University Hospital Jena, Salvador-Allende-Platz 27, 07747 Jena, Germany; 3 Department of Cardiothoracic Surgery, University Hospital Jena, Am Klinikum 1, 07747 Jena, Germany

**Keywords:** shock, ECMO, CytoSorb^®^, cytokine removal, ICU, acute heart failure, extracorporeal mechanical circulatory support

## Abstract

Background: Cardiogenic shock and arrest present as critical, life-threatening emergencies characterized by severely compromised tissue perfusion and inadequate oxygen supply. Veno–arterial extracorporeal membrane oxygenation (VA–ECMO) serves as a mechanical support system for patients suffering shock refractory to conventional resuscitation. Despite the utilization of VA–ECMO, clinical deterioration due to systemic inflammatory response syndrome (SIRS) resulting from the underlying shock and exposure of blood cells to the artificial surfaces of the ECMO circuit may occur. To address this issue, cytokine adsorbers offer a valuable solution by eliminating blood proteins, thereby controlling SIRS and potentially improving hemodynamics. Consequently, a prospective, randomized, blinded clinical trial will be carried out with ECMOsorb. Methods and Study Design: ECMOsorb is a single-center, controlled, randomized, triple-blinded trial that will compare the hemodynamic effects of treatment with a VA–ECMO in combination with a cytokine adsorber (CytoSorb^®^, intervention) to treatment with VA–ECMO only (control) in patients with cardiogenic shock (with or without prior cardiopulmonary resuscitation (CPR)) requiring extracorporeal, hemodynamic support. Fifty-four patients will be randomized in a 1:1 fashion to the intervention or control group over a 36-month period. The primary endpoint of ECMOsorb is the improvement of the Inotropic Score (IS) 72 h after the intervention. Prognostic indicators, including mortality rates, hemodynamic parameters, laboratory findings, echocardiographic assessments, quality of life measurements, and clinical parameters, will serve as secondary outcome measures. The safety evaluation encompasses endpoints such as air embolisms, allergic reactions, peripheral ischemic complications, vascular complications, bleeding incidents, and stroke occurrences. Conclusions: The ECMOsorb trial seeks to assess the efficacy of a cytokine adsorber (CytoSorb^®^; CytoSorbents Europe GmbH, Berlin, Germany) in reducing SIRS and improving hemodynamics in patients with cardiogenic shock who are receiving VA–ECMO. We hypothesize that a reduction in cytokine levels can lead to faster weaning from inotropic and mechanical circulatory support, and ultimately to improved recovery.

## 1. Introduction

In Europe, the yearly occurrence of cardiogenic shock resulting from cardiac arrest is estimated to range between 67 and 170 cases per 100,000 inhabitants [1]. When considering shock attributed to heart failure as well, the number rises to approximately 500,000 individuals [2]. However, poor overall survival persists, especially after cardiac arrest with a survival rate upon hospital discharge ranging from 0% to 18%, with an overall average of 8% [1].

The integration of percutaneous, mechanical, extracorporeal support for oxygenation and circulation serves as a secondary line of treatment for achieving hemodynamic stabilization [3,4] and veno-arterial extracorporeal membrane oxygenation (VA–ECMO) serves as a rescue therapy for patients experiencing shock that is unresponsive to conventional resuscitation efforts [3,5,6,7]. It functions as a bridging intervention, ensuring the maintenance of organ perfusion while the underlying cause of the shock is identified and addressed [3]. This treatment approach has demonstrated encouraging survival rates falling within the range of 20% to 30% [3,8,9].

Conditions that may necessitate ECMO support, such as cardiogenic shock or cardiac arrest, exhibit a shared pathogenetic mechanism of clinical deterioration, characterized by hyperinflammation [10,11,12]. This systemic inflammatory response syndrome (SIRS), which occurs to varying degrees in the majority of patients on ECMO support, is typically not infection-driven but rather a response to tissue injury associated with the underlying condition [12]. Furthermore, the ECMO support itself can trigger and sustain an inflammatory response through diverse mechanisms, including the contact between blood cells and molecules with the artificial surfaces of tubing, rotor, and oxygenator within the ECMO circuit [12,13]. Due to SIRS, the consequent development of multiorgan dysfunction syndrome (MODS) leads to a significant reliance on inotropic support. The administration of high doses and/or multiple different inotropes not only fails to alleviate tissue hypoxia and systemic inflammation but often exacerbates them, ultimately leading to increased mortality rates [12,14,15,16,17].

Extracorporeal hemoadsorption has emerged as a promising and innovative therapeutic approach to address this circumstance. Technological advancements have reached a stage where the properties of novel polymers can be harnessed for blood purification therapy. These polymers facilitate the selective and concentration-dependent removal of cytokines and other inflammatory mediators, offering a potential solution for targeted filtration [18,19]. CytoSorb^®^ (CytoSorbents Europe GmbH, Berlin, Germany) is a commercially available cytokine adsober which uses that concept [20]. Excessive levels of inflammatory mediators, including cytokines, interleukins, anaphylatoxins, as well as damage- and pathogen-associated molecular patterns, are irreversibly bound within the polymer through physico-chemical interactions [12].

In summary, the clinical necessity for interventions aimed at addressing inflammation and its cytokine storm is evident. Various strategies employed to manage the systemic inflammatory response through direct or indirect methods, such as steroids [12,21], plasma exchange [12,22], toll-like receptor antagonists [12,23], nitric oxide synthase inhibitors [12,24], or scavenger hemoglobin derivatives [12,25], have yielded conflicting or inconclusive outcomes, limiting their widespread application [12,26].

The ECMOsorb trial is designed to investigate the changes in hemodynamics, inflammatory biomarkers and immune system after implantation of a VA–ECMO in combination with an extracorporeal cytokine hemadsorption system.

## 2. Methods and Analysis

### 2.1. Study Design

**Figure 1:**
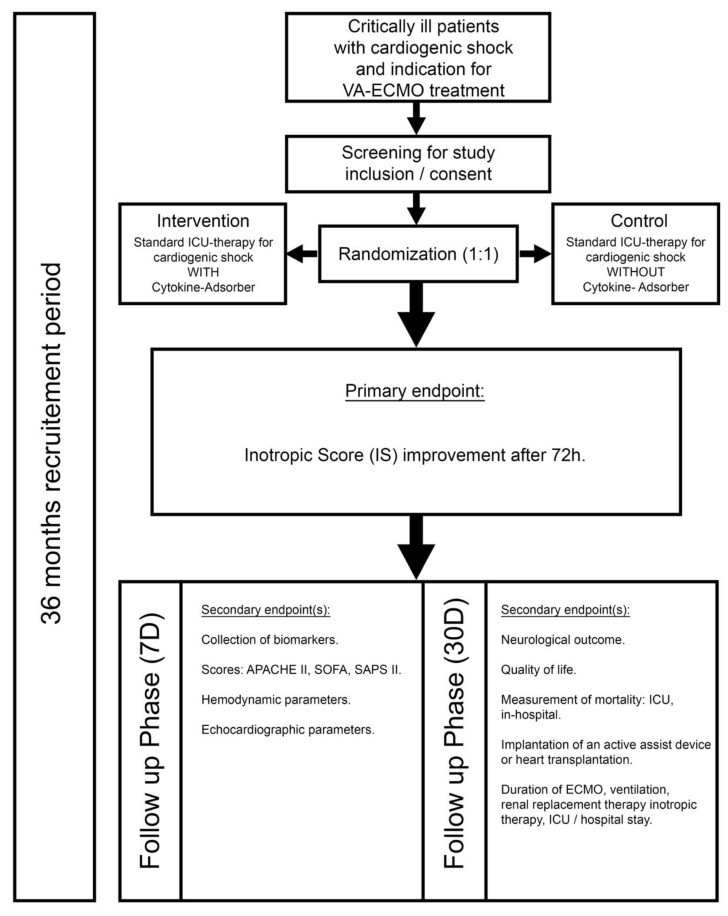
Flowchart of the study procedure. VA–ECMO = veno-arterial extracorporeal membrane oxygenation, ICU = intensive care unit, SAPS = Simplified Acute Physiology Score, APACHE = Acute Physiology and Chronic Health Evaluation, SOFA = sepsis-related/sequential organ failure assessment score, h = hours, D = days.

ECMOsorb is an interventional, prospective, two-arm randomized, controlled, triple-blinded trial. ECMOsorb will determine if the early, additional use of CytoSorb^®^ (CytoSorbents Europe GmbH, Berlin, Germany) is superior to standard ICU–treatment alone in patients with cardiogenic shock under VA–ECMO support. Figure 1 shows the flowchart of the trial. For patients in the interventional study arm, the CytoSorb^®^ hemadsorption device will be integrated into the VA–ECMO circuit within 6 h after ECMO implantation by a cardiac technician (perfusionist). The intervention phase is set for 72 h. The device will be changed every 24 h.

Each patient will be limited to a maximum of three adsorbers. Within the control group, patients will undergo VA–ECMO with the integration of a standard tube into the ECMO circuit, in place of the hemadsorption device. This integration task will be carried out by a proficient perfusionist.

**Figure 2:**
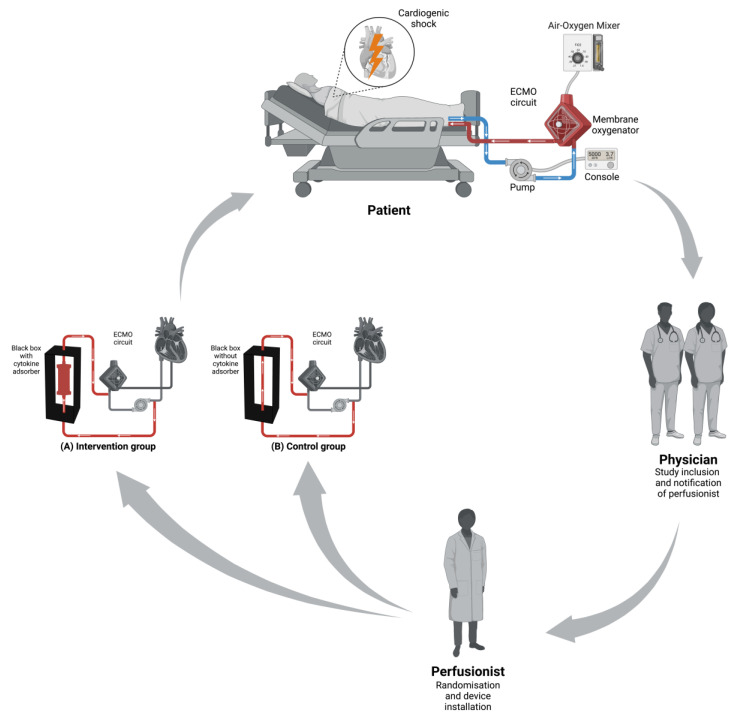
The schematic structure of the study inclusion and randomization process that lead to the two study groups. (**A**): intervention group with CytoSorb^®^ (=adsorber); (**B**): control group without CytoSorb^®^ (=tube). VA–ECMO = veno-arterial extracorporeal membrane oxygenation. Figure generated using BioRender^®^.

**Figure 3:**
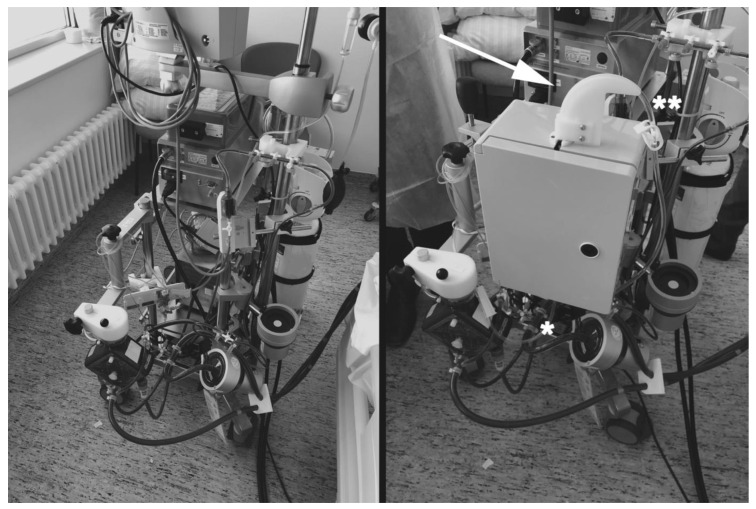
Left side: regular VA–ECMO (veno-arterial extracorporeal membrane oxygenation) machine set–up at the trial site from the manufacturer Getinge^®^ (Rastatt, Germany) that is part of the routine clinical use, including the BE–PLS 2050 Getinge^®^ (Rastatt, Germany), the Rotaflow RF–32 centrifugal pump (Getinge^®^, Rastatt, Germany), the QUADROX^®^ oxygenator (Getinge^®^, Rastatt, Germany) and standard tubing. The system is operated via the Rotaflow console (Getinge^®^, Rastatt, Germany) mounted on the Rotaflow Sprinter Unit with heater unit HU 35 (Getinge^®^, Rastatt, Germany) and manual blender. Right side: trial set up that includes the black box (CytoLock^®^ System, Jena, Germany) attached to the frame of the ECMO by a clamp mechanism. To avoid kinking of the tubing going in (*) and out (**) in the proximity of the box, a customized guiding contraption has been fitted (arrow, CytoLock^®^ System).

Regardless of group allocation, the hemadsorption device as well as the normal tube will be encapsulated by an always identically looking “black box” to ensure blinding (Figure 2 and Figure 3). Both groups will receive the VA–ECMO and standard of care for cardiogenic shock. Except for the adsorber, all other procedures and collected data will be identical in the two groups. An administration of the adsorber outside of this study for these patients will not take place. Only the perfusionist will be aware of treatment allocation. The interventionalist, the treating ICU team and the rest of the study team will thus be unaware of the group allocation. No interim analysis is planned or will be performed.

### 2.2. Study Population

Critically ill patients with cardiogenic shock with or without cardiopulmonary reanimation (CPR) and indication for VA–ECMO will be included.

### 2.3. Time Schedule, Study Duration and Frequency of Study Visits

The study is projected to span a period of 43 months, comprising a 36–month recruitment phase, a 1-month final follow-up period, and an additional 6 months dedicated to data analysis. The duration of intervention per patient is 72 h or termination of VA–ECMO (whichever occurs first). Follow-up per patient is 30 days after the beginning of the intervention. Table 1 outlines the study visits.

### 2.4. Screening and Randomization

All patients with cardiac shock requiring hemodynamic support via VA–ECMO referred to our department will be screened. Patients will be 1:1 randomized into one of the two groups.

### 2.5. Obtaining Informed Consent

Before inclusion, the nature, the aim and full extent of study participation will be explained to each subject, their legally authorized representative or an independent medical doctor, according to the Declaration of Helsinki to obtain informed consent.

### 2.6. Objectives

#### 2.6.1. Primary Objective

The primary objective of this study will be to demonstrate the benefit of a cytokine hemoadsorption device (CytoSorb^®^) compared to no device on hemodynamics of patients with cardiogenic shock under VA–ECMO treatment.

#### 2.6.2. Secondary Objective

The main focus of the secondary objectives will be to gain knowledge and study the mechanisms of subsequent prevention/limitation of the development of a cytokine-derived SIRS/multiple organ dysfunction syndrome (MODS) in these selected patients. Apart from laboratory parameters, the secondary objectives mostly include clinical surrogate measures (e.g., mortality) to confirm potential advantages of CytoSorb^®^ during the intervention and follow-up phase. Data acquisition during hospitalization and follow up is displayed in Table 1.

### 2.7. Endpoints

#### 2.7.1. Primary Endpoint

The primary endpoint of the ECMOsorb trial is the mean difference of the Inotropic Score (IS) 72 h after randomization (implantation of CytoSorb^®^ or a normal ECMO tube) between the two study arms. The Inotropic Score is calculated as: dopamine dose [μg/kg/min] + dobutamine dose [μg/kg/min] + 100xepinephrine dose [μg/kg/min] + 100× norepinephrine [μg/kg/min].

The role of dopamine in the management of cardiogenic shock remains a topic of debate. It has been observed that the administration of dopamine is associated with an increased incidence of arrhythmic events and higher mortality rates among patients with cardiogenic shock [27,28]. As a result, dopamine is not included in the repertoire of inotropic agents and vasopressors employed for the treatment of acute heart failure at our study site.

#### 2.7.2. Secondary Endpoints of the Study Are as Follows (Selection)

Duration of renal replacement therapy (continuous venovenous hemodialysis (CVVHD)), mechanical ventilation, ECMO therapy, inotropic/vasopressor treatment 7 and 30 days after the beginning of the intervention.

30–day mortality defined as mortality, in the hospital or anywhere after discharge, within 30 days after the beginning of the intervention.

Changes in mediators/markers of inflammation, myocardial damage, oxidative stress, myocardial wall stress, myocardial remodeling, kidney injury and kidney function at baseline, every 24 h during the intervention phase (up to 72 h) and on day 7 after the beginning of the intervention.

Length of stay in ICU and total length of hospital stay until discharge/transfer within 30 days after the beginning of the intervention.

Echocardiographic parameters of left and right ventricular function at baseline, every 24 h during the intervention phase (up to 72 h) and on day 7 after the beginning of the intervention.

Marker of hemolysis every 24 h under ECMO treatment (up to 72 h) after the beginning of the intervention.

Implantation of an active assist device or heart transplantation assessed on day 30 after the beginning of the intervention.

Simplified acute physiology score (SAPS) II, acute physiology and chronic health evaluation (APACHE) II; sequential organ failure assessment (SOFA) score at baseline, every 24 h during the intervention phase (up to 72 h) and on day 7 after the beginning of the intervention.

EQ–5D–3L questionnaire including EQ VAS (visual analogue scale), rehospitalization due to heart failure measured 30 days after the beginning of the intervention.

Neurological outcome: mRS (modified Rankin Scale), incidence of apoplexy, Glasgow Coma Scale (GCS), neuron-specific enolase (NSE), S–100, cerebral performance category (CPC), if clinically necessary and available: Computed tomography (head CT)/medianus somatosensory evoked potential (SEP) at baseline, every 24 h during the intervention phase (up to 72 h), on day 7 and on day 30 after the beginning of the intervention.

### 2.8. Inclusion Criteria

Critically ill patients with cardiogenic shock with or without reanimation (CPR) and indication for VA–ECMO with the following mandatory requirements will be included: Systolic blood pressure < 90 mmHg, >30 min of inotropic agents and/or vasopressors to keep the blood pressure > 90 mmHg systolic, signs of left ventricular failure with pulmonary congestion and signs of end organ hypoperfusion (at least one of the following criteria: clouding of consciousness, cold, pale skin or extremities, oliguria (≤30 mL/h), serum lactate > 2.0 mmol/L, age ≥ 18 years ≤ 80 years). In order to be enrolled in the study, participants are required to provide a signed informed consent document.

### 2.9. Exclusion Criteria

Exclusion criteria are: contraindications to VA–ECMO implantation, patients with pre-existing sepsis (raised C-reactive protein (CRP), positive procalcitonin (PCT), leukocytosis, fever, positive blood cultures), shock duration > 12 h before evaluation, severe PVD (peripheral vessel disease) making ECMO–implantation impossible, aortic valve regurgitation/stenosis at least II°, age: <18, >80 years, CNS (central nervous system) disease with fixed, dilated pupils (not drug-induced), severe concomitant disease with limited life expectancy < 6 months, participation in another study, cardiopulmonary resuscitation (CPR) > 60 min, shock due to other reasons, pregnancy, HIT (Heparin-induced thrombocytopenia) positive, very low platelet counts (<20,000/μL), body weight less than 45 kg and a current immunosuppressive or immunomodulatory therapy.

### 2.10. Technology Used in the Trial

#### 2.10.1. ECMO

For the ECMOsorb trial, patients with cardiogenic shock requiring mechanical, circulatory support in form of VA–ECMO will be recruited. ECMO is a method of extracorporeal organ replacement in which the pulmonary and/or the cardiac function are partially or completely replaced. VA–ECMO in particular drains venous blood from a large–lumen vein near the right atrium through a cannula and connected tubing system and returns it via inflow into the arterial system, typically to the iliac arteries toward the aorta. Cannulas are inserted percutaneously, usually by Seldinger technique, through which blood is conveyed by means of a centrifugal pump through a membrane oxygenator and returned to the patient. During the circuit passage, the blood runs through an extracorporeal gas exchange unit where it is oxygenated, decarboxylated and warmed. ECMO represents a massive right–left shunt establishing flow rates of up to 10 L per minute depending on cannula and rotor type. The clinical result is a significant increase in blood pressure in the presence of adequate vascular resistance.

At the trial site, an ECMO from the manufacturer Getinge^®^ (Rastatt, Germany) will be used. This artificial circuit (Figure 3) consists of a preassembled, standard tubing set (BE–PLS 2050, Getinge^®^) that includes the Rotaflow RF–32 centrifugal pump (Getinge^®^, Rastatt, Germany), QUADROX^®^ oxygenator (Getinge^®^, Rastatt, Germany) and standard tubing. The system is operated via the Rotaflow console (Getinge^®^, Rastatt, Germany) mounted at the Rotaflow Sprinter Unit with heater unit HU 35 (Getinge^®^, Rastatt, Germany) and manual blender. The circuit will be established via complete lower body cannulation of femoral vessels using sheath sizes 17–21 Fr (arterial) and 19–25 Fr (venous). All patients receive an antegrade limp perfusion via an additional 7 Fr bypass cannula (CruraSave^®^ Femoral–Perfusion Set, free life medical GmbH^®^, Aachen, Germany). If additional left ventricular (LV) support is required to address persistent, insufficient cardiac output, implantation of a percutaneous microaxial pump (Impella CP^®^, Abiomed, Danvers, MA, USA) from a femoral arterial access to facilitate LV venting will be performed. An intraaortic balloon pump (IABP) will not be used.

#### 2.10.2. VA–ECMO Weaning

Initiating weaning from VA–ECMO involves an individualized strategy that requires hemodynamic stability, an improvement of left ventricular pump function and a controlled underlying cause of cardiogenic shock. Patients experiencing ongoing cardiac failure are deemed unable for weaning. A daily evaluation to assess the potential for weaning is implemented using clinical, laboratory, and echocardiographic data that either favor weaning or may be suggestive for difficulties in weaning. Criteria for initiating a weaning attempt include (selection): a left ventricular ejection fraction (EF) of at least 20%, pulsatile flow, normalized arterial lactate, a mean arterial pressure of at least 65–70 mmHg, low requirement of vasopressor or inotropic support, oxygen saturation nearly 95%, central venous oxygen concentration >60% and a low ventilatory support for normal lung ventilation and oxygenation while on VA–ECMO support. Weaning is initiated by gradually reducing VA–ECMO pump flow while monitoring cardiac function continuously. If signs of inadequate cardiac output or tissue perfusion, e.g., increasing blood lactate levels, are observed, the patient will be returned to higher VA–ECMO support. Once a minimal VA–ECMO support (<2 L per minute) has been established during the weaning process, ensuring hemodynamic stability, termination of the extracorporeal circulation will take place and the cannulas will be clamped. Immediate decannulation using access site closure devices such as Angioseal^®^ (TERUMO^®^, Somerset, NJ, USA) and/or ProGlide^®^ (Abbott Park, IL, USA) will be performed at the bedside for percutaneously placed cannulas. Central cannulation or surgical cutdown requires cannula removal in the operating room. After complete and successful weaning, continuous monitoring of cardiac output is necessary to ensure organ perfusion is maintained. Additional ongoing inotropic support may be required for certain patients. Patients unsuitable or unable for weaning will be considered for transplant or assist device implantation (LVAD).

#### 2.10.3. Cytokine Adsobers

**Figure 4:**
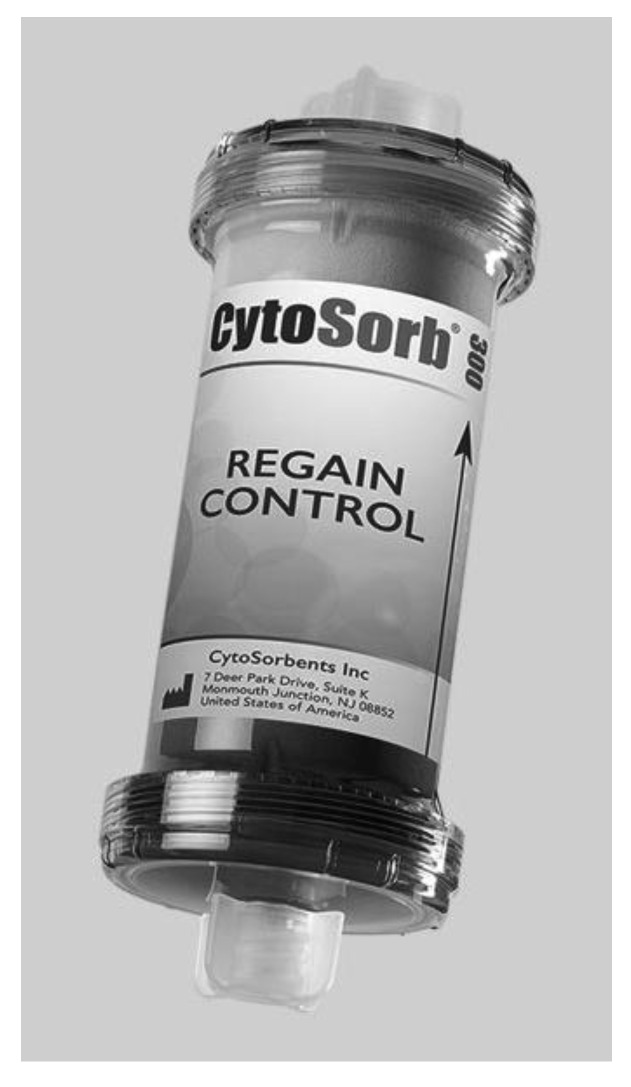
The study device: Cytokine adsorber CytoSorb^®^ (with kind permission from CytoSorbents^®^ Europe GmbH, Berlin, Germany).

The adsorbers that are currently in clinical use have already received CE–approval for medical devices [12]. In this study the CytoSorb^®^ adsorber (CE 0344, CytoSorbents Europe GmbH, Berlin, Germany) serves as medical device for the intervention group (Figure 4). CytoSorb^®^ is ISO 10993 biocompatible, meeting strict standards of hemocompatibility and biocompatibility, and is compatible with both systemic heparin and regional citrate anticoagulation. The CytoSorb^®^ is a single-use device and will not be reused. The functioning of cytokine adsorbers is very similar to a hemoperfusion therapy [12]. In detail, it is an extracorporeal cartridge made of a highly porous, bio-compatible, polystyrene-based polymer beads with a mean diameter of 450 μm each [12]. The total polymer surface is thus extremely large (>45,000 square meters) [12]. The cartridge is perfused with whole blood [12]. Each 300 mL cartridge is pre-filled with a sterile isotonic sodium chloride solution [12]. Within the polymer, excessive levels of inflammatory mediators, such as cytokines, interleukins, anaphylline toxins, as well as damage- and pathogen-associated molecular patterns, are irreversibly bound in a physico-chemical manner [12]. The removal of these substances is concentration–dependent, so that low (physiological) cytokine levels are not affected [12]. Mainly hydrophobic (water-insoluble) molecules can be removed up to a size of 55 kDa, a size range that covers most pro–inflammatory mediators, but remains non–specific [12,29]. The service life of a cartridge is up to 24 h and the adsorber is approved for blood flow rates between 100 and 700 mL/min, while a blood flow of at least 150 mL/min is recommended [12]. For the priming and integration of Cytosorb^®^ into the ECMO circuit, commercial sets are available. After purging the cytokine hemoadsorber with saline solution and establishing an activated partial thromboplastin time (aPTT) of 60–80 s/activated clotting time (ACT) of 160–210 s, a portion of blood will then be branched off from the ECMO tubing system right before passing through the oxygenator. It will then flow through the cartridge and be guided into the ECMO outflow cannula of the patient before entering the pump. These connector sets are designed to be cut off from the ECMO circuit in order to replace the cartridge after 24 h of use without any changes to the ECMO circuit. The extracorporeal circuit and the additional circuit for the adsorber/tube will be monitored continuously.

#### 2.10.4. Black Box

The “black box” used to establish blinding, is a professionally manufactured plastic box with metal components capable of encapsulating either the adsorber cartridge (intervention) or a regular tube in case of the control group (CytoLock^®^ System, Jena, Germany, Figure 2 and Figure 3). This plastic box meets all the hygiene requirements of our ICU and does not cause any dysfunction of the ECMO circuit. Tubing going in and out of the box are identical in length, appearance and cannot be pushed in or pulled out regardless of group allocation. To avoid kinking of the tubing in the proximity of the box, a customized guiding contraption has been fitted.

### 2.11. Safety, Possible Complications, Risks

Study participation will not be associated and/or expected with any additional risks for a patient’s safety.

The safety of the adsorber will be evaluated on both clinical and device–based parameters. Clinical parameters will include the occurrence of (severe) adverse device effects, the monitoring of vital signs, and clinical laboratory changes. Device-specific parameters will include the incidence of any device related technical issues that may adversely affect treatment, e.g., critical changes in pressure profiles of the extracorporeal circuit, that may be caused by obstructions, leaks or breaks, or any other physical failures of the device. The adsorber will not be used if it appears to be damaged, and if beads appear to be free floating within the endcaps.

Patients will be monitored for clinical events associated with extracorporeal treatment. Documentation of relevant events regarding the adsorber (CytoSorb^®^) includes but is not limited to: periprocedural complications within 30 days combined from (pericardial effusion, stroke, major bleeding, embolization, intracranial bleeding), device related (thrombus, breakage, malfunction, allergic reactions), air embolism, technical and procedural success of device implantation and severe hypotension.

Events which are not plausibly explainable by the underlying disease (cardiogenic shock) or a known comorbidity or if the event is considered or suspected to have a causal relationship to the adsorber are classified as adverse events. The definitions are based on the new Medical Device Regulation (EU) 2017/745.

In compliance with Article 2, Number 58 of the Medical Device Regulation (MDR), a serious adverse event (SAE) refers to any adverse event that resulted in one or more of the following outcomes: death; serious deterioration in the subject’s health leading to a life-threatening illness or injury, permanent impairment of a body structure or function, hospitalization or prolonged hospitalization, medical or surgical intervention to prevent life-threatening illness or injury, or permanent impairment of a body structure or function; and the development of a chronic disease.

It should be noted that planned hospitalization for a pre-existing condition or a procedure required by the study protocol, without any serious deterioration in health, is not considered a serious adverse event. These criteria ensure the comprehensive assessment and reporting of adverse events throughout the study.

Occurrences will be systematically documented from the time of study inclusion and throughout the follow–up period, which extends up to day 30 after the commencement of implantation. In case of an emergency within the intervention phase (first 72 h after implantation) that requires the knowledge of the treatment arm of the patient, the black box will be opened, and the respective device will be removed if an adverse effect of the adsorber is suspected. In case an unblinding is needed after the 72 h intervention phase has passed, an unblinding list will be used to find out in which arm the patient is classified. The integrity of all bloodlines and connections prior to the initiation and periodically during the treatment will be checked by a perfusionist and by the treating ICU team. To avoid any kind of air embolism, we use a priming set for the cartridge.

### 2.12. Statistical Considerations and Methods

#### 2.12.1. Sample Size

Sample size calculation was conducted for the primary outcome of Inotropic Score (IS) at 72 h following CytoSorb^®^ or tube implantation. The estimated sample size is based on the standard deviation (SD) of the inotropic score after 72 h from our own VA–ECMO patient population (SD = 5.6). To detect a difference of 5 points in the IS score between both groups with a power of 80%, a total of 44 patients (21 per group) need to be analyzed (two-sided independent samples t test, α = 0.05). Considering an anticipated dropout rate of 20%, the trial aims to recruit 54 patients, with 27 patients assigned to each group.

#### 2.12.2. Statistics

The primary outcome analysis will involve comparing the experimental group receiving CytoSorb^®^ with the control group receiving standard care (without CytoSorb^®^), focusing on the differences in the mean Inotropic Score (IS) at 72 h post-implantation. The primary analysis will encompass the full analysis set, including all patients who were randomized and received treatment, following the intent-to-treat (ITT) principle. Sensitivity analyses are planned in the per-protocol (PP) analysis set. A linear model will be fitted for the primary endpoint with treatment and baseline IS as fixed effects. The confirmatory analysis will be performed at a significance level of 5%. The mean difference of the primary endpoint will be estimated within the model and will be reported with 95% confidence interval. Categorical endpoints will be compared by Chi-square test/Fisher’s exact test between groups. According to the data distribution, two-sided independent samples *t* test or Mann–Whitney U test will be applied for continuous endpoints. Adequate descriptive statistics will be used to summarize the data in each group. All secondary analyses will be performed exploratively, i.e., without adjustment for multiplicity. Safety analyses will be run in the safety population to be defined in the protocol; these analyses will summarize and tabulate all observed safety events including a measure of uncertainty (i.e., 95% confidence intervals).

### 2.13. Addressing Bias in the Methods

To account for both recognized and unrecognized predispositions and potential prognostic factors, and to mitigate or eliminate possible systematic variations resulting from varying levels of expertise, a rigorous approach involving triple-blinding and randomization will be implemented.

#### 2.13.1. Randomization

1:1 block randomization will be performed centrally by means of an online tool (PaRANDies^®^, Jena, Germany). Following the randomization process, patients who are assigned to either of the compared treatments are included in the full analysis set, which adheres to the principles of the intention-to-treat analysis.

#### 2.13.2. Treatment Bias

This study incorporates a triple-blinded design and evaluates treatment strategies in real-world settings. Concealment of study treatment allocation from patients and the medical staff will be achieved by using the black box. As a final step for data analyses, the statistician is also blinded. Additionally, our research center possesses a certified specialized cardiac ICU dedicated to the management of cardiac shock and the implementation of ECMO techniques. These practices are integrated within a comprehensive quality management system. The investigators have received training in good clinical practice (GCP). All healthcare professionals involved possess the necessary qualifications, including national licenses, or operate under the supervision of senior staff members.

#### 2.13.3. Measurement Bias

During each visit, potential outcomes will be recorded and documented in the designated case report forms (CRFs). Furthermore, follow-up will be conducted for all patients after the screening process, randomization, and implantation (with CytoSorb^®^ or without CytoSorb^®^). Finally, the trial will be monitored by the Department of Cardiology and the Center for Clinical Studies at Jena University Hospital, ensuring oversight throughout the study.

### 2.14. Data Management

#### 2.14.1. Data Assessment/Case Report Forms

Data documentation is conducted utilizing a web-based study management software (OpenClinica^®^, Waltham, MA, USA) as case report form (CRF), facilitating efficient and comprehensive data capture and management. The software complies with regulatory requirements such as Good Clinical Practice (GCP, 21CFR Part11). Data entry will be performed using web browser input masks with an encrypted connection (HTTPS) to ensure secure transmission. To ensure pseudonymized data analysis, a unique and unambiguous patient identification number will be assigned to each participant. This approach guarantees the confidentiality and privacy of the subjects’ information during the analysis process. The data undergo verification through range, validity, and consistency checks. In cases of missing or implausible data, the study team receives a request for clarification, ensuring data quality and integrity throughout the study. Any modification of data in the electronic case report form will be documented in an automated audit trail. Any unauthorized data access is prohibited by a hierarchical system that assigns specific user roles.

#### 2.14.2. Ethics Approval

The study was approved by ethics committee of the Friedrich Schiller University Jena, Germany (No.: 2020–2027_1–BO) in December 2020.

#### 2.14.3. Privacy, Collection and Processing of Data

All data and information gathered throughout the study will be handled in strict compliance with the comprehensive regulations outlined in the national data protection law (EU-DSGVO). The utmost confidentiality and appropriate measures for data usage and protection will be ensured, maintaining the privacy and security of the collected information. Throughout the study, participants will be exclusively identified using individual identification codes (subject number). To ensure the protection of this data, comprehensive organizational procedures have been implemented to prevent unauthorized access or distribution of the data.

#### 2.14.4. Dissemination

The results of this study will be published in a reputable international medical journal, irrespective of the findings obtained.

## 3. Discussion

Despite the growing utilization of ECMO in various life-threatening acute conditions [12,30,31,32], significant progress in the field of mechanical circulatory support and critical care, the rates of morbidity and mortality among ECMO patients continue to be alarmingly high [12]. The ICU mortality rate for our ECMO patients is approximately 80%.

The effectiveness of CytoSorb^®^ remains a subject of controversy [33]. Triple-blinded, randomized trials investigating the acute hemodynamic, echocardiographic, immunological, and cellular effects of VA-ECMO with the concurrent use of an adsorber to mitigate or potentially prevent an associated inflammatory response in patients with cardiogenic shock have not yet been conducted. ECMOsorb aims to assess the effectiveness of CytoSorb^®^ through a triple-blinded, randomized trial involving a larger cohort of patients with cardiogenic shock and VA-ECMO. The trial will focus on evaluating a clinical endpoint associated with hemodynamic improvement. Establishing efficacy would represent an initial evidence-based advancement for this challenging patient population that is often difficult to treat.

## Figures and Tables

**Table 1:** Frequency and scope of ECMOsorb study visits.

Protocol Items	Time Point						
			S	V0	V1	V2	V3
	Routine Exami-nation	Informed Consent Process	Screening	Baseline	Intervention	Post Intervention	Post Intervention
		Day 0–30		Day 0	0–72 h	7 Days after Intervention	30 Days after Intervention
**Trial related procedures**							
Inclusion/exclusion criteria			X				
Informed consent		X					
Patient registration				X			
Randomization				X			
Study device ^†^ initiation				X ^1^	X ^2^		
**General examination**							
Medical history, comorbidities ^∆^	X		X				
Physical examination ^∆^	X		X	X			
Prior/concomitant medication ^∆^	X		X	X			
Neurological exam (incl. mRS) ^∆^	X		X	X	X	X	X
Quality of Life (EQ -5D-3L including EQ VAS)						X	X
SCOREs (SAPS II, APACHE II, SOFA) ^∆^	X			X	X	X	
Adverse events/complications ^∆^				X *	X	X	X
**Diagnostic procedures**							
12-lead surface ECG ^∆^	X		X	X	X		
TTE ^∆^	X		X	X	X	X	
Technical data from ECMO ^∆^	X			X	X	X	
Laboratory evaluation ^∆^	X		X	X	X	X	
Local blood chemistry ^∆^	X		X	X	X		
PAC–Hemodynamics ^∆^	X			X	X		
Measurement of parameters of hemodynamics ^∆^	X		X	X	X	X	
Measurement of mortality ^∆^	X					X	X
Measurement of duration of ECMO, ventilation, dialysis, inotropic therapy ^∆^	X			X	X	X	X
Measurement of the neurological outcome ^∆^	X			X	X	X	X
VA–ECMO Implantation	X			X			

^∆^ Data documentation from clinical routine (if available). * Recording of AEs/SAEs after implantation of CytoSorb® adsorber or ECMO tube, ^†^ device = either CytoSorb^®^ adsorber (= intervention group) or ECMO tube (= control group) via black box. ^1^ within the first 6 h after implantation of VA-ECMO, ^2^ changed every 24 h within the intervention of 72 h, V = visit, mRS = modified Rankin Scale, SAPS = Simplified Acute Physiology Score, APACHE = Acute Physiology And Chronic Health Evaluation, SOFA = sepsis-related/sequential organ failure assessment score, ECG = electrocardiography, TTE = transthoracic echocardiogram, ECMO = Extracorporeal membrane oxygenation, PAC = pulmonary artery catheterization, VAS = visual analogue scale, h = hours.

## Data Availability

The data to be collected for this study will not be publicly available due to local legal restrictions on data safety and privacy. However, in exceptional cases, access to the study raw data may be granted to authorized researchers or institutions after obtaining appropriate approvals and ensuring data anonymization and confidentiality.
